# A Real-Time FPGA Implementation of Infrared and Visible Image Fusion Using Guided Filter and Saliency Detection

**DOI:** 10.3390/s22218487

**Published:** 2022-11-04

**Authors:** Ling Zhang, Xuefei Yang, Zhenlong Wan, Dingxin Cao, Yingcheng Lin

**Affiliations:** 1 The School of Microelectronics and Communication Engineering, Chongqing University, Chongqing 400044, China; 2 National Information Center of GACC, Beijing 100005, China

**Keywords:** image fusion, saliency detection, multi-scale decomposition, FPGA

## Abstract

Taking advantage of the functional complementarity between infrared and visible light sensors imaging, pixel-level real-time image fusion based on infrared and visible light images of different resolutions is a promising strategy for visual enhancement, which has demonstrated tremendous potential for autonomous driving, military reconnaissance, video surveillance, etc. Great progress has been made in this field in recent years, but the fusion speed and quality of visual enhancement are still not satisfactory. Herein, we propose a multi-scale FPGA-based image fusion technology with substantially enhanced visual enhancement capability and fusion speed. Specifically, the source images are first decomposed into three distinct layers using guided filter and saliency detection, which are the detail layer, saliency layer and background layer. Fusion weight map of the saliency layer is subsequently constructed using attention mechanism. Afterwards weight fusion strategy is used for saliency layer fusion and detail layer fusion, while weight average fusion strategy is used for the background layer fusion, followed by the incorporation of image enhancement technology to improve the fused image contrast. Finally, high-level synthesis tool is used to design the hardware circuit. The method in the present study is thoroughly tested on XCZU15EG board, which could not only effectively improve the image enhancement capability in glare and smoke environments, but also achieve fast real-time image fusion with 55FPS for infrared and visible images with a resolution of 640 × 470.

## 1. Introduction

Interestingly, imaging complementarity of infrared and visible light imaging has attracted increasing interest for image fusion. It is well-established that visible light sensor can obtain high-resolution images with abundant texture and detailed information, but the quality of images captured by visible light sensor is strongly affected by the light environment, as poor illumination will reduce the visual image quality such as glare, smoke, overexposure, and so on. Compared to visible light imaging, infrared imaging is less affected by the illumination conditions and has strong penetrability to glare, smoke, etc. Images captured by infrared sensor could provide thermal information for the target and offer high contrast, but they lack the texture information and sensitivity of the non-thermal target. Therefore, it is anticipated that infrared and visible image fusion can cooperatively combine the merits of individual imaging technologies while minimizing potential defects.

With the increasing demands of infrared and visible image fusion technology for applications such as video surveillance, autopilot, military reconnaissance, etc., there has been a surge in the number of fusion methods that can be roughly divided into three categories: pixel level fusion, feature level fusion, and decision level fusion [1]. Pixel level-based fusion is the most common fusion method at present, which can be roughly summarized as multi-scale decomposition (MSD), sparse representation (SR), deep learning (DL), saliency detection (SD), and hybrid methods.

The MSD-based method is achieved through decomposing the source image into different layers to represent the various spatial and frequency domain information of the source image, then applying specific fusion rules to different layers to obtain the corresponding fusion layer, and finally reconstructing the fused sub-layers to obtain the fused image. To sum up, the central challenge of MSD-based methods is the decomposition and fusion of images [2]. Pyramid transform was the earliest methodology applied for image fusion applications, and the typical approaches of pyramid transforms are the Laplacian pyramid [3,4], contrast pyramid [5], etc. However, the image fusion methods based on pyramid transform will introduce halo artifacts. To reduce halo artifacts, some wave transforms were recently applied to optimize infrared and visible image fusion, such as wavelet transform [6], discrete wavelet transform [7], dual-tree discrete wavelet transform [8], curvelet transform [9], contourlet transform [10], etc. Although the wave transform-based method can practically reduce halo artifacts, it cannot preserve the edge of the source image in the complex spatial structure. Consequently, edge preservation filter technique was later proposed and applied for image processing such as bilateral preservation filter, guided filter, rolling guided filter [11,12,13,14], etc., which can effectively preserve the edge of the source image in the process of image decomposition. For example, Li et al. [15] proposed an image fusion algorithm based on the guided filter. Bavirisetti et al. [16] proposed a multi-scale decomposition rule-based guided filter to obtain the different layer sublayer by iterative. Liu et al. [17] use guided filter combined with other methods to construct fusion weight map, which can enhance the detail and edge information for the fused image. Shreyamsha Kumar et al. [18] proposed an image fusion method based on cross bilateral preservation filter combining the similarity and spatial structure of local areas. Lin et al. [19] proposed a multi-scale decomposition method using rolling guided filter. In addition to the above methods, anisotropic heat diffusion [20], log-Gabor transform [21], etc. have also been successfully integrated to improve the quality of MSD-based image fusion.

Saliency is an attention mechanism that can attract human visual perception, and its key merit is that it is more efficient to capture human visual senses than the neighborhood of the point or the areas [22]. Saliency detection can focus on the region of interest, so it is very suitable for the field of image fusion. The SD-based method is usually not used alone but combined with multi-scale decomposition for image fusion. The implementation of saliency detection is carried out sequentially: (1) decomposing source image into detail layer and base layer and (2) using saliency detection to process detail or base layer. Durga Prasad Bavirisetti et al. [22] use the average filter and median filter to extract saliency information and construct a fusion weight map when performing detail layer fusion. Duan et al. [23] use local average gradient energy to extract multiple saliency features on infrared and visible detail layers, which can enhance the detail information performance of the fused image. Lin et al. [24] proposed a saliency detection rule based on local brightness contrast to extract the saliency layer which contains brightness contrast information of the source image. Besides these methods mentioned above, some other image fusion methods [25,26] also use saliency detection to improve the expressiveness of the fused image. These studies collectively supported that SD-based method can maximize focus on regions of interest.

Besides the methods mentioned above, SR-based, DL-based, and hybrid methods [27,28,29,30,31,32,33,34,35,36,37] are also reported for infrared and visible image fusion. Specifically, for the SR-based methods, an over-complete dictionary is used to represent the source image, and the correlation coefficients obtained from the over-complete dictionary are used for fused operation. However, the over-complete dictionary is often difficult to acquire, and a single over-complete dictionary is usually insufficient to ensure the robustness of images with different structures. With the development of the deep learning, the DL-based method has been a popular model in the field of image fusion. For instance, Li et al. proposed three infrared and visible image fusion methods using different neural network models, which is VGG19 [27], ResNet [29] and DenseFuse [30], respectively. These methods can not only autonomously extract image features and fit suitable fusion coefficients but also get the good quality for fused image. However, the fused image weak the thermal target information and lack the visual detail information. In order to improve the image details and maintain the edge information, Lui et al. proposed a novel method based on ResNet and rolling guided filter [31]. Although DL-based method has some advantages, the training process requires large amount of training data and is very time-consuming, leading to low computational efficiency. The hybrid methods [35,36,37] can yield fused images with good quality by exploiting complicated compute models, but their computational efficiency is also very low.

Because of its parallel computing capacity, FPGA has become a promising implementation carrier for computational acceleration. Currently, many researchers have done a lot of work for image fusion based on FPGA to improve image fusion speed. For instance, various methods based on pyramid transform [38,39] and wave transform [40,41] are widely implemented on FPGA to meet real-time requirements, but these methods can introduce the artifacts and lack the edge structures relatively. In addition, Furkan Aydin et al. implement an image fusion method on FPGA using high-level synthesis (HLS) tools and other technologies based on color space transformation, mean, and variance [42] to improve the image’s color information, Ashutosh Mishra et al. implement an image fusion method based on two-scale decomposition using average filter and fuse the detail layer using modified Frei-Chen operator [43]. These methods can achieve image fusion and get the good process speed on FPGA, but the performance of the fused image would be reduced and have influence of glare, smoke, etc. restricted by the fusion model.

According to the introduction mentioned above, the time consumption of the image fusion process for those advanced algorithm is too long to meet real-time requirements, and the fused image is affected by glare, smoke, etc. which will reduce the performance of the fused image. To solve these problems, this paper proposes an MSD-based image fusion algorithm using guided filter and saliency detection that has high affinity for hardware. This method decomposes source image into three-scale layers while preserving the edge structure and construct the saliency layer fusion weight map using attention mechanism to eliminate the influence of the glare, smoke, etc. on the fused image. At the same time, the paper uses HLS to design, test and verify the method based on FPGA, which can accelerate the processing of the fusion method to meet the real-time requirements. Compared to many advanced fusion methods, this method can enhance performance of the fused image in the case of as low computational complexity as possible and eliminate the effects of the glare, smoke, etc. Apart from this, due to the simplification of computational operations, this method is more suitable to use hardware to accelerate process.

Section 2 and Section 3 will introduce the detailed fusion method and analysis the fusion result. The FPGA implementation of this method will be introduced in Section 4.

## 2. Proposed Fusion Method

### 2.1. Overview of Guided Filter

Guided filter [13], an edge preservation filter, is originally proposed by K. M. He et al., which mainly performs smoothing operations by considering the statistical characteristics of the neighboring pixels of the target pixel. The output calculated by the guided filter is the same as syn-linear time-invariant filter [16]. When performing the smoothed process, the correlation will be excited between the source image and the guided image by guided filter, which can preserve the information for specific regions. Same as other edge-preserving filters, the guided filter can preserve edge during image decomposition, which helps to avoid ringing artifacts. In addition to the edge-preserving property, the guided filter could also offer structure-transfer property. When the guided image is the same as the input image, the edge-preserving smooth operation is performed by the guided filter that will retain the structural behavior of the input image. When the guided image is different from the input image, the structural behavior of the output image smoothed by the guided filter will be affected by the guided image. Based on the above factors, when the infrared and visible images are decomposed, the mutual guidance in between will improve the structural characteristics of decomposed images and enhance the fusion effect. The steps of the guided filter are summarized in Algorithm 1.

**Algorithm 1:** Algorithm of guided filter
**Input: guided image *I*, input image *p*, radius *r*, regularization *eps***

**Output: smoothed image *q***
1:

$mean\_I=f_{ave}\left(I,r\right)$



$mean\_p=f_{ave}\left(p,r\right)$



$mean\_II=f_{ave}\left(I.\times I,r\right)$



$mean\_Ip=f_{ave}\left(I.\times p,r\right)$

2:

cov_Ip=mean_Ip−mean_I.×mean_p



var_I=mean_II−mean_I.×mean_I

3: a=cov_Ip./(var_I+eps)
b=mean_p−a.×mean_I
4: $mean\_a=f_{ave}\left(a,r\right)$

$mean\_b=f_{ave}\left(b,r\right)$
5: q=mean_a.×I+mean_b$f_{ave}$: average filter process

At the same time, compared with the edge-preserving filter such as the cross bilateral filter, the rolling guided filter, etc., the computation of the guided filter is smaller and simpler, so it is easier to be implemented into hardware. The specific calculation strategy of the guided filter can be summarized as shown in Algorithm 1. More detailed information about the guided filter, please reference [13].

### 2.2. Image Decomposition Strategy

In this paper, the smoothing process by the guided filter can be described using the following formula:

(1) $$q=f_{GF}(I,p,r,eps)$$

$f_{GF}$ is the guided filtering function. The means of *I*, *p*, *r*, *eps*, and *q* are consistent with those summarized in Algorithm 1.

#### 2.2.1. Source Image Decomposition

Based on the scope of this study, we comprehensively describe the methods for the decomposition of infrared and visible light images. In this multi-scale decomposition method, the source image is firstly decomposed into the base layer and detail layer using the guided filter, the specific operation can be summarized as follows:

(2) $$I_{vis}^{b}=f_{GF}(I_{ir},I_{vis},r,eps)$$

(3) $$I_{ir}^{b}=f_{GF}(I_{vis},I_{ir},r,eps)$$

$I_{vis}$ and $I_{ir}$ represent the visible source image and infrared source image, respectively. $I_{vis}^{b}$ and $I_{ir}^{b}$ represent the visible base layer and infrared base layer, respectively. In our implementation, *r* = 7, *eps* = 1000. To obtain the detail layer of the source image using the guided filter, the base layer is subtracted from the source image. This operation can be summarized as follows:

(4) $$I_{i}^{d}=I_{i}-I_{i}^{b},i=vis,ir$$

$I_{vis}^{d}$ and $I_{ir}^{d}$ are the visible detail layer and infrared detail layer, respectively.

After the above operation, the source image is successfully decomposed into the detail layer and base layer, of which detail layer contains the small detailed structure, edge, and texture information, while the base layer contains the brightness contrast information, background information, etc., in the source image.

#### 2.2.2. Base Layer Decomposition

The human visual perception system can easily perceive regions that are significantly different from their neighborhoods in a large amount of data, during which those data were labeled as salient information from the source data. Based on the saliency theory, the saliency detection method can extract the complex saliency information of the infrared and visible light images using different saliency detection rules and use it to improve the expressiveness of the fused image. To reduce the influence of the glare, smoke and other detrimental factors on the fused image, it is necessary to perform saliency detection in the base layer containing brightness contrast information via decomposing the base layer to obtain the saliency layer. Herein, we used the attention mechanism to solve the effect of glare, smoke, etc. in the fused image when performing saliency layer fusion. The following formulae describe the method used in the present study to extract the saliency layer from the base layer, and the extraction process can be summarized as follows:

(5) $$S_{i}(x,y)=|\bar{B_{i}}-\frac{1}{N}\sum_{j\in \Omega_{N}}B_{i}(x,y)|,i=vis,ir$$

(6) $$S_{{}_{i-norm}}(x,y)=\frac{S_{i}(x,y)-\min(S_{i}(x,y))}{\max(S_{i}(x,y))-\min(S_{i}(x,y))},i=vis,ir$$

Firstly, according to the brightness contrast rule, the local mean value $(\sum_{j\in \Omega_{N}}B_{i}(x,y))/N$ centered at pixel (x,y) is subtracted from the global mean value $\bar{B_{i}}$ of the base layer, and then operate the absolute operation for the value calculated above to obtain the saliency weight map $S_{i}(x,y)$. To extract the saliency layer, a normalized operation is performed by saliency weight map on the obtained saliency weight map, where local areas represent the windows areas with a radius of N × N (N = 7 for the implementation in this study). |∙| represents the operation of calculating the absolute value. After obtaining the extraction coefficient $S_{{}_{i-norm}}(x,y)$ of the saliency layer, the base layer can be decomposed into the saliency layer and background layer. The steps can be summarized in the following expressions:

(7) $$I_{i}^{s}(x,y)=I_{i}^{b}(x,y)\times S_{{}_{i-norm}}(x,y),i=vis,ir$$

(8) $$I_{i}^{bg}(x,y)=I_{i}^{b}(x,y)-I_{i}^{s},i=vis,ir$$

Then, the base layer is decomposed to saliency layer $I_{i}^{s}(x,y)$ and background layer $I_{i}^{bg}(x,y)$. According to the imaging principle, the saliency layer separated by this method contains the area of interest, such as areas of high brightness contrast—glare, smoke, etc. areas in the visible image and thermal target regions in the infrared image. The background layer contains the rough background information.

### 2.3. Image Fusion Strategy

#### 2.3.1. Detail Layer Fusion

The “maximum absolute value” strategy is commonly used for detail layer fusion, which has a low computational cost and can maintain the edge and detail information, but its anti-noise performance is usually unsatisfactory that may often introduce noise and artifacts in the fused image. Therefore, to achieve balanced performance of computational complexity, anti-noise performance and fusion effect, we adopt a weight fusion strategy when performing the detail layer fusion. At the same time, considering that the detail layer will be affected by noise, instead of directly using the pixel value of the original detail layer as the fusion weight, the filtered detail layer by an average filter with a kernel size of 7 × 7 is used as the detail layer fusion weight. The fusion coefficient of the detail layer will be constructed using the following expression:

(9) $$W_{i}^{d}(x,y)=\frac{|I_{i}^{d\_blur}(x,y)|}{|I_{vis}^{d\_blur}(x,y)|+|I_{ir}^{d\_blur}(x,y)|},i=vis,ir$$

$I_{i}^{d\_blur}(x,y)$ represents the infrared or visible detail layer processed by the average filter. $W_{i}^{d}(x,y)$ represents the fusion weight coefficient of the infrared or visible detail layer. When the fusion weight was obtained, the fused detail layer $I_{F}^{d}(x,y)$ can be obtained using the following expression:

(10) $$I_{F}^{d}(x,y)=I_{vis}^{d}(x,y)\times W_{vis}^{d}(x,y)+I_{ir}^{d}(x,y)\times W_{ir}^{d}(x,y)$$

#### 2.3.2. Saliency Layer Fusion

Since the high-brightness areas in the saliency layer represent the regions of interest that contain different meanings. The common regions of interest in visible light image, the regions of interest represent glare, smoke and other areas. Meanwhile, thermal target areas are the common regions of interest for infrared image. The regions of interest in the infrared and visible saliency layers are mutually exclusive. On the one hand, the regions of interest in the visible saliency layer should be suppressed to reduce the effects of the fused image. On the other hand, the regions of interest in the infrared saliency layer should be maintained or enhanced to improve the performance of the thermal target in the fused image. Hence, how constructing the fusion weight coefficient of saliency layers is very important. Equation (11) describes the process of constructing saliency layer fusion weight map.

(11) $$\begin{array}{l} if(\bar{I_{{}_{vis}}^{s}}<\frac{1}{N}\sum_{j\in \Omega_{N}}I_{{}_{vis}}^{s}(x,y))\\ \;\;\;\;\;\;\;\;\;\;\;M_{vis}^{s}(x,y)=I_{ir}^{s}(x,y)\\ \;\;\;\;\;\;\;\;\;\;\;M_{ir}^{s}(x,y)=I_{vis}^{s}(x,y)\\ elseif(\bar{I_{{}_{ir}}^{s}}<\frac{1}{N}\sum_{j\in \Omega_{N}}I_{{}_{ir}}^{s}(x,y)\&\&\bar{I_{{}_{vis}}^{s}}<\frac{1}{N}\sum_{j\in \Omega_{N}}I_{{}_{vis}}^{s}(x,y))\\ \;\;\;\;\;\;\;\;\;\;\;M_{vis}^{s}(x,y)=(I_{ir}^{s}(x,y)+I_{vis}^{s}(x,y))\times (1-\alpha )\\ \;\;\;\;\;\;\;\;\;\;\;M_{ir}^{s}(x,y)=(I_{ir}^{s}(x,y)+I_{vis}^{s}(x,y))\times \alpha \\ else\\ \;\;\;\;\;\;\;\;\;\;\;M_{vis}^{s}(x,y)=I_{vis}^{s}(x,y)\\ \;\;\;\;\;\;\;\;\;\;\;M_{ir}^{s}(x,y)=I_{ir}^{s}(x,y) \end{array}$$

Specifically, regions where the local mean of the saliency layer $(\sum_{j\in \Omega_{N}}I_{i}^{s}(x,y))/N$ is greater than the global mean $\bar{I_{i}^{s}},i=vis,ir$ will be identified as the regions of interest. If the region of interest is detected only in the visible saliency layer, the corresponding pixels of the infrared and visible layer will be exchanged to construct the fusion weight map; if the same regions of interest are detected in the visible and infrared saliency layers, the fusion weight value to construct the fusion weight map will be distributed according to coefficient α, whereas the coefficient α should be greater than 0.5 (α = 0.8 in our implementation). Besides those areas mentioned, the other areas’ original pixel value of the infrared and visible saliency will be directly used for constructing the fusion weight map. This processing strategy of the saliency layer can overcome the influence of glare, smoke, etc., and enhance the thermal target in the fused image.

The fusion weight map of the saliency layer constructed by this rule will be affected by the noise and suffer from the brightness-edge effect. To overcome those problems, an average filtering operation with an average filter kernel size of 15 × 15 will be performed for the fusion weight map. After smoothing for fusion weight map of saliency layer, using the normalization operation to obtain the final fusion weight coefficient.

(12) $$W_{i}^{s}(x,y)=\frac{|M_{i}^{s\_blur}(x,y)|}{|M_{vis}^{s\_blur}(x,y)|+|M_{ir}^{s\_blur}(x,y)|},i=vis,ir$$

where $M_{i}^{s\_blur}(x,y)$ represents the smoothed saliency layer using average filter, $W_{i}^{s}(x,y)$ represents the final fusion weight coefficient. After obtaining $W_{i}^{s}(x,y)$, the fusion operation of the saliency layers will be performed according to the following expression:

(13) $$I_{F}^{s}(x,y)=I_{vis}^{s}(x,y)\times W_{vis}^{s}(x,y)+I_{ir}^{s}(x,y)\times W_{ir}^{s}(x,y)$$

$I_{F}^{s}(x,y)$ is the fused saliency layer. According to the above fusion rules, the fused saliency layer eliminates potential detrimental factors such as smoke and glare in the visible light saliency layer and maintains or highlights the thermal target information in the infrared channel.

#### 2.3.3. Background Layer Fusion

The background layer contains some relatively rough information, and its entropy is at a low level, so the weight average fusion strategy is used in performing the background layer fusion to obtain the fused background layer $I_{F}^{bg}(x,y)$, which can reduce the amount of necessary computation. The operation of the background layer fusion can be summarized as follows:

(14) $$I_{F}^{bg}(x,y)=(I_{vis}^{bg}(x,y)+I_{ir}^{bg}(x,y))\times 0.5$$

### 2.4. Image Reconstruction and Enhancement

After obtaining the fused detail layer, fused saliency, and fused background layer, the final fused image is obtained by the hierarchical integration of the three different scale layers. The process of image reconstruction can be expressed as follows:

(15) $$I_{F}^{}(x,y)=I_{F}^{d}(x,y)+I_{F}^{s}(x,y)+I_{F}^{bg}(x,y)$$

$I_{F}(x,y)$ represents the fused image of the infrared and visible light images, which contains the image information therein and may address the interference of glare, smoke, etc. while enhancing the thermal target performance in the fused image.

Image enhancement technology can achieve operations such as changing the image contrast and enhancing the detailed information, so the rational implementation of image enhancement technology can effectively improve the expressiveness of the image. Due to the low resolution of the infrared image and poor imaging environment, the contrast of the fused image is usually not good in general. Herein, an image enhancement method using the guided filter was executed for the fused image to improve its contrast. This method can multiplex modules during hardware design, thereby reducing design complexity. The steps of the image enhancement can be described using the following expressions:

(16) $$I_{F}^{b}=f_{GF}(I_{F},I_{F},r,eps)$$

(17) $$I_{F}^{d}=I_{F}-I_{F}^{b}$$

(18) $$I_{F}^{enh}=I_{F}^{d}\times \beta +I_{F}^{b}$$

$I_{F}^{b}$ and $I_{F}^{d}$ represent the base layer and detail layer of the fused image, respectively, $I_{F}^{enh}$ represents the final output image via this infrared and visible image fusion method. *β* represents the enhancement factor. In our implementation, the values of *r*, *eps*, and *β* were 7, 1000, and 3, respectively. This image enhancement method could substantially improve the edge, detailed information, and contrast of the fused image, which can enhance the expressiveness of the fused image in terms of visual perception.

## 3. Algorithm Experiment and Analysis

For the experiment and analysis of the algorithm, images from commonly used public datasets as well as self-made datasets for testing the performance. Public datasets include TNO-dataset [44] and CVC-14 dataset [45]. The selected test datasets contain various scenes, which can comprehensively challenge the above algorithm. In this study, the proposed method will be comparatively analyzed with five advanced infrared and visible image fusion algorithm—MDLatLRR [46], MGFF [16], MSID [47], ResNet [29], and RGFF [19].

### 3.1. Subjective Analysis

Subjective evaluation is mainly based on the intuitive feeling of the human visual system, for which the region of interest can quickly be distinguished by vision and attention while useless information is discarded in the fused image. In the following analysis, the glare, smoke, and normal dark scenes will be selected for analysis.

**Figure 1:**
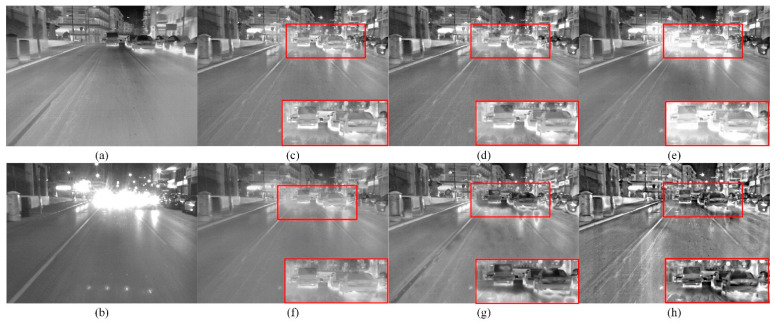
Fused images in the glare scene using six different methods. (**a**) Infrared image; (**b**) Visible image; (**c**) MDLatLRR; (**d**) MGFF; (**e**) MSID; (**f**) ResNet; (**g**) RGFF; (**h**) Proposed.

Figure 1 shows the fused images in the urban road scene with glare influence using six different fusion methods, of which the glare area is marked with red rectangles. It is observed that all the six methods have completed the fusion task. The fused images obtained by MDLatLRR, MGFF, and ResNet methods in the glare scene preserve the detailed information while reducing the impact of glare, but the contrast is low and the target information is not prominent. The fused image based on the MSID method in the glare scene is pronouncedly affected by the glare factor and shows overexposure in the red box area, and the targets cannot be clearly detected in the fused image. The fused image using the RGFF method in this scene has good performance and the targets are clear in the glare area. However, from the overall expressiveness of the image, sharp brightness edges are present in the red box, thus reducing the visual perception. Compared with the other five fused images, the fused image using the proposed method is not affected by the glare factor, and the targets in the red box area are salient. In addition, the integral performance of the fused image shows greater benefit under high contrast.

**Figure 2:**
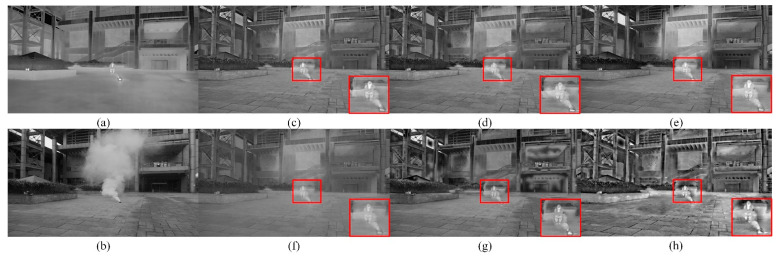
Fused images in the smoke scene using six different methods. (**a**) Infrared image; (**b**) Visible image; (**c**) MDLatLRR; (**d**) MGFF; (**e**) MSID; (**f**) ResNet; (**g**) RGFF; (**h**) Proposed.

Figure 2 shows the fused images in the outdoor scene with smoke influence using six methods mentioned above. It can be seen from Figure 2 in the smoke scene that the six methods all show considerable fusion performance to some extent. The fused image based on the MGFF method is seriously affected by smoke and less capable of preserving the thermal target. The fused image obtained via RGFF method is less affected by the interference of smoke but the contrast is relatively lower. Similar issues have also been found for the fused image via ResNet method, which lacks detailed information, and the thermal target is not clear. Compared with the four fusion methods mentioned above, the fused image by the MDLatLRR method and the proposed method are not affected by smoke, while the proposed method shows significantly higher contrast than the MDLatLRR method, validating its superior visual perception capacity.

**Figure 3:**
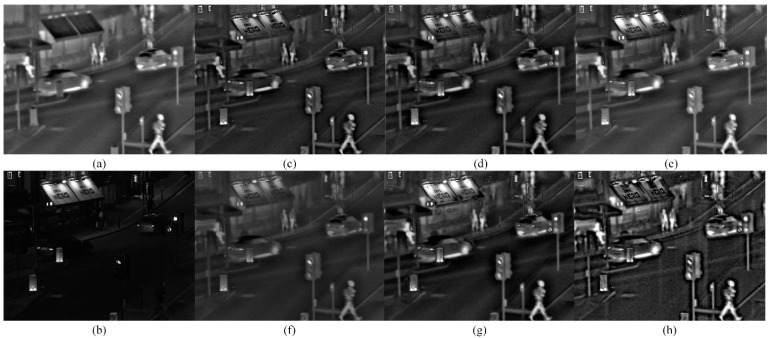
Fused images in the normal dark scene using six different methods. (**a**) Infrared image; (**b**) Visible image; (**c**) MDLatLRR; (**d**) MGFF; (**e**) MSID; (**f**) ResNet; (**g**) RGFF; (**h**) Proposed.

Figure 3 shows the fused images obtained by six fusion methods in the normal dark scene. Since infrared imaging does not require extra illumination, it is widely applied under dark environment. According to the results in Figure 3, the thermal target in the fused image obtained by the MDLatLRR method and the MGFF method is good but not prominent. The fused image by the ResNet method lacks detailed information and is very blurry, leading to poor recognition of the thermal target and contrast. Overall, the MSID method, RGFF method, and proposed method showed roughly identical performance in preserving the thermal target in the fused image is roughly equivalent. Those three methods mentioned above enhance the thermal imaging performance for selected targets, but the contrast of the fused image obtained by the proposed method is visibly better than the others.

**Figure 4:**
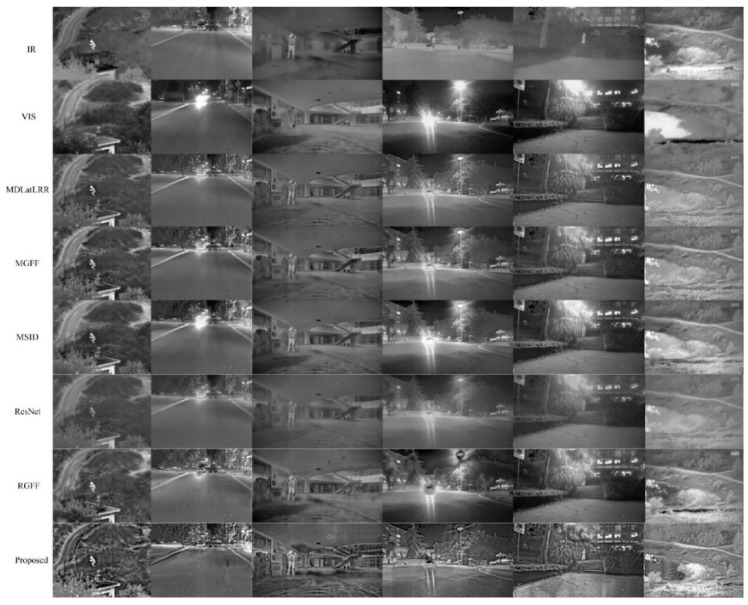
The other six pairs of fused images obtained by six different methods.

In addition to the above scenes, the six methods have been thoroughly analyzed in a comparative manner under different scenes, which contain the wild scene and lake scene on the day, the campus scene with glare at night, and indoor scene with smoke. Figure 4 shows the effects of the results obtained by six different fusion methods, which immediately suggests that the performance of the proposed method in this study is superior to the other established methods.

### 3.2. Objective Analysis

After subjective evaluation, we employed the objective method to analyze the nine pairs of the images mentioned in subjective analysis because of the high persuasiveness of the indexes. The objective indexes include entropy (EN) based on information theory, average gradient (AG), and edge intensity (EI) based on image features, structural similarity index measure (SSIM) based on the similarity between fused image and source image, visual information fidelity, information fusion (VIFF) based on visual perception. From these five indexes, the comprehensive evaluation is carried out using VIFB benchmark [48].

Table 1 shows the average values of five metrics for nine pairs of infrared and visible fused images obtained by six methods for objective evaluation of their performance in different scenes, which can reflect the general applicability of the proposed method in complex environment. The five evaluation metrics selected are all positively correlated with the expressiveness of the fused image. It is observed that the proposed method we developed presents the highest AG, EN, EI, and VIFF values, indicating its capability to enhancing the expressiveness of the fused image via integrating image information, preserving the edge and improving visual perception. However, the metric of SSIM for the fused image obtained by the proposed method is not very prominent compared to those established methods, and the possible explanation is that the reason is that the method we developed would alter the image structure when performing contrast enhancement and thus reduce SSIM performance, which is a metric characterizing the structural similarity between the fused image and the infrared and visible light source images.

Due to limitation of hardware, it is necessary to reduce the time consumption of the fusion process to facilitate hardware implementation. This test software is MATLAB R2019, the hardware parameters of the PC platforms are as follows: intel core i7-7700HQ CPU, 8 GB memory, and Nvidia GeForce 1050 graphics. Table 2 shows the results of the average time-consumption of the six fusion methods for processing 9 pairs of infrared and visible light images. Although the average time consumption of the image fusion process by the proposed method is slightly longer than MSID, it is significantly more effective than the other methods. The proposed method is based on the pixel level method, which is suitable for dataflow optimization operation in hardware implementation and accelerate the computation.

The comprehensive subjective and objective evaluation shows that the method we developed can achieve good fusion image expressivity with low time consumption and is convenient for hardware implementation, thus presenting high practical value for various real-life applications.

## 4. Hardware Implementation

### 4.1. Design of FPGA Implementation

The hardware implementation of the infrared and visible image fusion is designed by the Vavido HLS tool, which can convert high-level language (C/C++) to hardware design language such as Verilog or VHDL, thus shortening the development cycle of FPGA. At the same time, this tool has good support for the floating-point operation, which is friendly for the image fusion process that requires relatively high calculation accuracy.

**Figure 5:**
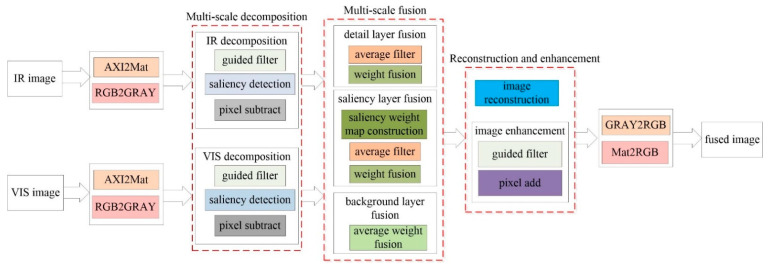
Schematic illustration for the top design of the image fusion module.

Figure 5 shows the scheme of the image fusion module design for the proposed method based on FPGA implementation. In this design, the AXI interface is used as the data transport interface between the processing system (PS) and programming logic (PL). In the design of the PL region, the conversion of AXI and MATRIX data formats is implemented using the official library API, and the remaining modules mainly include image decomposition, image fusion, and image reconstruction and enhancement. The specific implementation procedures of individual modules are described by the processing flow chart chapter 2.

**Figure 6:**
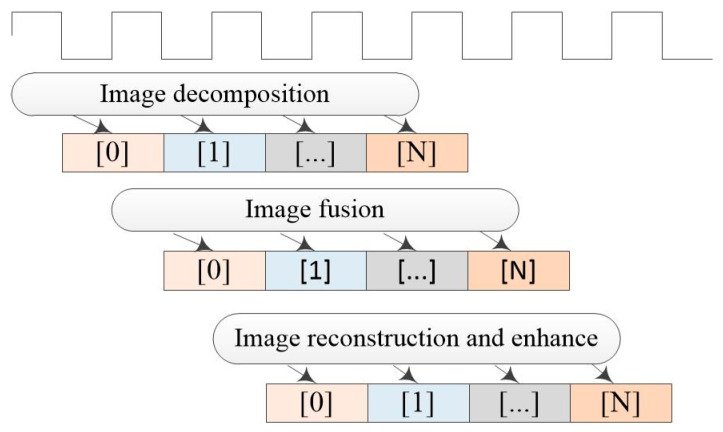
Parallelized operation with dataflow pragma.

For the purpose of computational optimization, the dataflow pragma is used to parallelize the data processing. After the optimization treatment, the FIFO buffer will be generated to store the data between the processing stages; therefore, achieving streamlined computation. In addition, the pipeline pragma can unroll the loop to achieve the parallel process. These optimization pragmas are suitable for the image fusion method as each pixel is processed independently, which can speed up the image fusion process and achieve real-time image output. Figure 6 shows the parallelized operation with dataflow in the top design, through which the single pixel processed by the current stage will be stored in the FIFO buffer and read by the next stage in an individualized manner, which is not affected by the processing status of other pixels. The dataflow pragma and pipeline pragma are also used in sub-module design in a similar manner, thus realizing the streamlined and parallelized processing of images and reducing the consumption of the storage resources.

**Figure 7:**
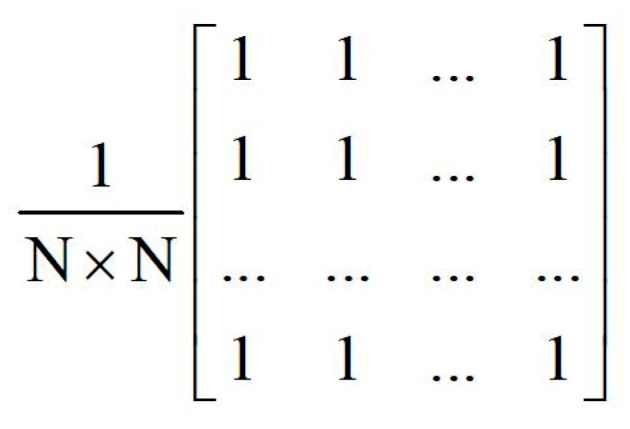
Average convolution operator of size N × N.

The average filter is used repeatedly when performing the image fusion to smooth the image or calculate the local mean, so it is important to rationally design this module. The calculation of the average filter is based on the average convolution operator shown in Figure 7. However, this strategy would require large amount of computation and would increase exponentially with the increasing size of the average filter kernel. To solve this problem, we adopts the box filter method with O(1) computational complexity to reduce the amount of calculation that is not affected by N.

**Figure 8:**
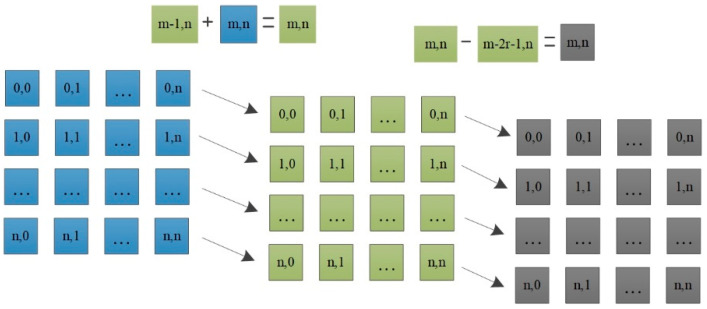
Calculation rule based on the columns of the box filter.

The procedures of the box filtering include columns operation and row operation, which have the same calculation rule. Figure 8 shows the calculation rule based on columns. First, the columns of the original pixel are summed, and the position of the corresponding pixel is updated to the sum of the topmost pixel value of the column for the corresponding pixel. Subsequently, according to the column sum obtained after the update, the local column sum of length N is obtained. After the column-based operation is completed, the local sum of columns as the original value is subjected to a row operation same as the column operation and then normalized to achieve average filter.

### 4.2. FPGA Implementation Result

The hardware implementation is designed, tested, and verified based on the XCZU15EG board of the FPGA board AXU15EG series. The resolution of the infrared and visible light images is 640 × 470 in our implementation. In the design process, the data accuracy is preserved as much as possible, which can prevent the excessive loss of data accuracy and the resultant impairment of expressiveness for the fused image.

**Figure 9:**
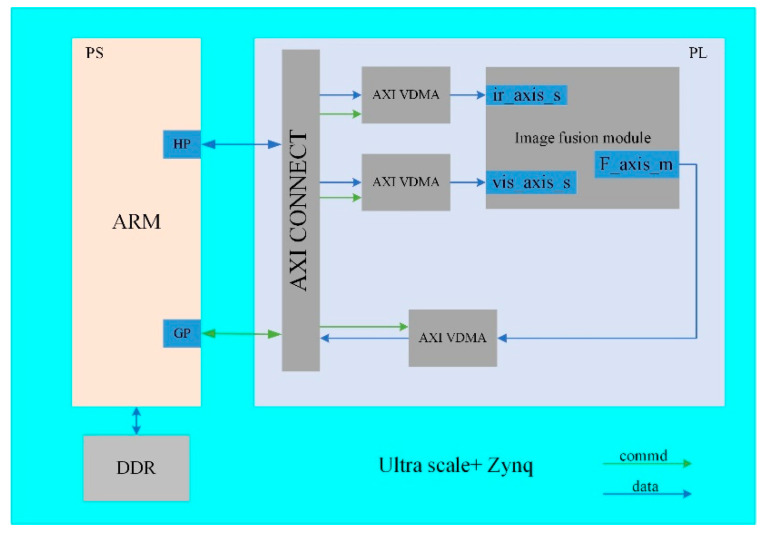
System block diagram for the FPGA implementation.

Table 3 shows the resource consumption of this developed image fusion module based on FPGA implementation. After synthesis and simulation, a test platform is built based on ARM+FPGA Architecture in Figure 9. The AXI video direct memory access (AXI VDMA) is used to read infrared and visible source images and store fused image. The HP interface is used for data interaction between ARM and AXI VDMA, and the config commands are sent to VDMA through GP interface.

**Figure 10:**
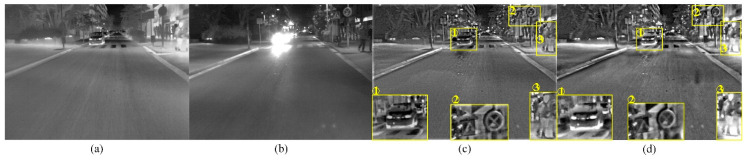
Test result in the glare scene. (**a**) Infrared image; (**b**) Visible image; (**c**) Fused image on FPGA; (**d**) Fused image on PC.

**Figure 11:**
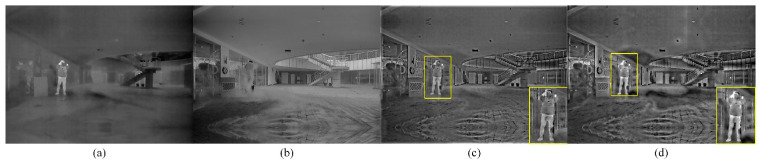
Test result in the smoke scene. (**a**) Infrared image; (**b**) Visible image; (**c**) Fused image on FPGA; (**d**) Fused image on PC.

Table 4 shows the processing speed based on FPGA and PC. As can be seen from Table 4, the processing speed of the image fusion module is about 18 ms (55FPS) under the 100 MHZ reference clock. Compared to the processing speed implemented on PC, the speed of processing on FPGA is roughly 63 times faster, leading to almost real-time output of the fused image. The test results of the fused images on FPGA are shown in Figure 10 and Figure 11.

Figure 10 shows the fused images obtained on FPGA and PC under the glare environment. From the perspective of the overall expressiveness, the performance of the fused images obtained on the FPGA and PC is both acceptable, although certain differences exist due to a certain loss of precision in hardware processing. Compared with the fused image obtained on the PC, the brightness contrast of the fused image on the FPGA has decreased, which leads to lower contrast and clarity of the targets in the yellow block 1 and yellow block 3. For the yellow block 2 in Figure 10a,b, the traffic signs are not significantly different. Based on the analysis of the three important areas and the overall expressiveness of the fused image, it could be concluded that the image fusion process based on FPGA successfully reduced the glare effects in the fused image and achieved image fusion despite slight decrease in expressiveness.

Figure 11 shows the fused images obtained on FPGA and PC under the smoke environment. In this scene, the brightness contrast of the fused image obtained on FPGA is lower than that obtained on PC, similar to performance in the glare scene. The yellow rectangle areas in Figure 11a,b where the thermal target is located, respectively, the expressiveness of the thermal target has decreased processed by FPGA than PC in the yellow rectangle area. Besides, the detail and texture information of the fused image obtained on FPGA suffered a slight loss. This is explained by the loss of accuracy after the hardware implementation, which causes the insensitivity to some low brightness or not-highlight detailed area when performing the image fusion process. To sum up, after hardware-based implementation, the fusion performance is within acceptable range despite a slight decrease in the quality of the fused image in the smoke environment.

Table 5 shows the average metrics of the fused images obtained using different platforms in the above two test datasets. According to the objective analysis in Table 5, AG, EN, EI, VIFF of the fused image obtained on FPGA have decreased compared with that obtained on PC, but the SSIM has slightly improved. The result of the objective analysis is consistent with the subjective performance of the fused image in Figure 10 and Figure 11, where the fused image obtained on FPGA showed slight loss in the detail and texture information as well as decreasing brightness contrast, resulting in attenuated overall expressiveness of the fused image. Based on the above analysis, the image fusion speed through hardware implementation is 63 times higher than that of the PC, leading to balanced performance among fusion speed, image quality, data indicators and visual experience.

**Figure 12:**
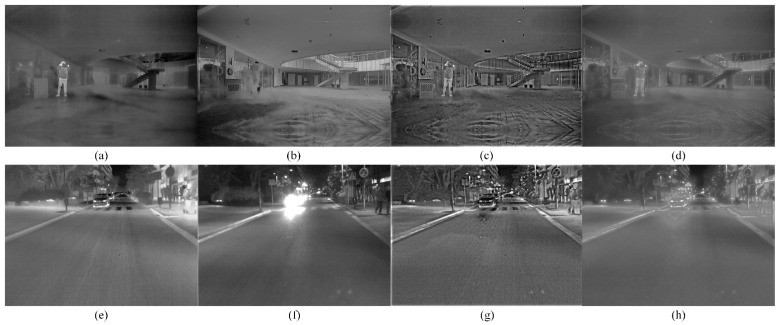
Subjective comparison of the fused image based on FPGA. (**a**) Infrared image with smoke; (**b**) Visible image with smoke; (**c**) Fused image obtained by proposed method in the smoke scene; (**d**) Fused image obtained by Modify-Frei-Chen operator based method in the smoke scene; (**e**) Infrared image with glare; (**f**) Visible image with glare; (**g**) Fused image obtained by proposed method in the glare scene; (**h**) Fused image obtained by Modify-Frei-Chen operator based method in the glare scene.

Figure 12 shows the comparative result of the two FPGA-based methods (Modified-Frei-Chen operator-based and proposed) in the two scenes. According to the result, the fused images obtained by Modified-Frei-Chen operator-based method in two scenes have common characteristic compared with the proposed method. The common feature is that lack some details and edges, and where the thermal target is not prominent. In the meantime, the car, traffic sign and other targets of interest to visual perception are not clear because of the low contrast. From the aspect of the subjective analysis, although the fused images are not affected by glare, smoke, etc. obtained by two methods, the overall performance of the fused image obtained by proposed method is better than another mentioned. Table 6 shows the objective analysis result for two methods in the two scenes, compared with the Modify-Frei-Chen operator (MFCO) based method, the five metrics of the proposed method are better.

## 5. Discussion

At Present, quality and speed are still huge challenges in the field of infrared and visible image fusion. To enhance the performance of the fused image, enormous complex methods are proposed and make progress in this aspect, but the speed of the fused image is reduced because of complex computations, and simpler fusion models cannot guarantee the quality of the fused image. How to get the balance between the quality and speed for image fusion to meet real-time requirement is important.

In view of the slow fusion speed, most researchers implement algorithms based on FPGA to speed up the image fusion process. At present, the mainstream FPGA-based algorithms mainly focus on pyramid transform, wave transform and multi-scale transform. Their fusion effect is relatively poor and cannot highlight salient regions, which is not conducive to image fusion for regions of interest.

In this paper, a hardware-friendly infrared and visible light image fusion method based on guided filter and saliency detection is developed exploiting FPGA as the hardware circuit, which presents a viable solution for real-time scenarios such as automatic driving, video surveillance, military reconnaissance, etc. Compared with other advanced infrared and visible light image fusion methods, the computation and complexity of the proposed method are significantly lower. At the same time, this method eliminates the effects of the glare, smoke, etc., to the fused image. This paper analyzes the quality of the fused images based on PC, the experimental results show that the fused images obtained by the proposed method have good expressiveness, and the time consumption is about 1.15 s for each image with a resolution of 640 × 470. Although this is still insufficient for real-time image processing, it is still much faster than the other advanced image fusion method. To accelerate the processing speed, we designed a hardware circuit based on FPGA by exploiting its parallel computation capacity, which can enhance the processing speed to about 18 ms per image, thus realizing real-time output of the fused image. The FPGA-based image fusion method may offer balanced performance between fusion speed and image quality, which shows promise for real-life applications.

## Figures and Tables

**Table 1:** Objective analysis for different methods.

	AG	EN	EI	SSIM	VIFF
MDLatLRR	4.3380	6.7158	43.4766	1.5001	0.5611
MGFF	4.4433	6.8275	44.5341	1.4840	0.6935
MSID	4.1946	7.0509	42.1197	1.4815	0.5924
ResNet	2.7246	6.4840	26.4826	1.5363	0.3367
RGFF	4.4842	6.9844	45.3183	1.4201	0.4831
Proposed	7.7220	7.1230	77.1950	1.1486	0.7701

**Table 2:** Average time consumption of the six fusion methods.

	MDLatLRR	MGFF	MSID	ResNet	RGFF	Proposed
Time/s	63.73	2.39	0.34	6.67	19.77	1.29

**Table 3:** FPGA resource requirements.

Resource	Used	Available	% of All
BRAM_18K	740	1488	49
DSP48	391	3528	11
FF	97,624	682,560	14
LUT	143,594	341,280	42

**Table 4:** Speed performance compared to PC implementation.

FPGA Maximum Clock Frequency	100 Mhz
FPGA Maximum Frame Rate	55 fps
PC/MATLAB R2019b (i7-7700HQ @ 2.80 GHz)	0.87 fps
speedup	63×

**Table 5:** Objective analysis for two fused images obtained on FPGA and PC.

	AG	EN	EI	SSIM	VIFF
FPGA	5.5974	6.5457	56.4529	1.4671	0.6218
PC	6.1449	6.7678	61.3421	1.3007	0.7383

**Table 6:** Objective analysis for two FPGA-based fusion method in the two scenes.

	AG	EN	EI	SSIM	VIFF
MFCO-based	1.5360	6.0851	16.1062	1.6318	0.2280
Proposed	5.5974	6.5457	56.4529	1.4671	0.6218

## Data Availability

The data presented in this study are available on request from the corresponding author. The data are not publicly available due to privacy restrictions.
